# Encopresis in Children: A Report of 20 Cases

**DOI:** 10.7759/cureus.84951

**Published:** 2025-05-28

**Authors:** Abir Azirar, Amal Hamami, Maria Rkain

**Affiliations:** 1 Department of Pediatrics, Faculty of Medicine and Pharmacy, Mohammed VI University Hospital, Mohammed I University, Oujda, MAR; 2 Department of Pediatric Gastroenterology, University Hospital Center (CHU) Mohammed VI, Oujda, MAR

**Keywords:** chronic constipation, encopresis, fecal incontinence, pediatrics, polyethylene glycol

## Abstract

Introduction: Encopresis, or fecal incontinence, is a distressing condition in children over the age of four characterized by the repeated passage of stool in inappropriate places. It is often associated with chronic constipation and can have significant psychosocial impacts on affected children and their families.

Objective: The objective of this study was to describe the epidemiological, clinical, and therapeutic characteristics of children aged four years and older diagnosed with encopresis and followed in a pediatric gastroenterology outpatient clinic, with particular attention to distinguishing secondary encopresis in children who have previously achieved bowel continence.

Methods: A retrospective descriptive study was conducted using data from HOSIX hospital software (SIVSA, Spain). All pediatric patients diagnosed with encopresis, regardless of etiology, and seen in consultation during 2021 and 2024 were included.

Results: The mean age of symptom onset was five years. Most cases (80%, N = 16) occurred after the age of five, with no instances of primary encopresis identified. The majority of patients (70%, N = 14) were from urban settings. Chronic constipation was the leading cause (85%, N = 17), followed by neurological etiologies (10%, N = 2) and a single case of congenital megacolon. The average delay between symptom onset and initiation of treatment was 12 months. Treatment strategies primarily involved polyethylene glycol (PEG)-based laxatives, dietary modifications, and behavioral interventions, with favorable outcomes in most cases.

Conclusion: Encopresis is a prevalent condition in pediatrics, typically secondary to chronic constipation. Early diagnosis and a multidisciplinary management approach are crucial for effective treatment. Delayed care remains a challenge and may contribute to the chronicity.

## Introduction

Encopresis, or fecal incontinence, is defined as the repeated passage of stool in inappropriate places (e.g., underwear), whether involuntary or intentional, in children aged four years or older who have already achieved toilet training [1]. According to the Diagnostic and Statistical Manual of Mental Disorders, Fifth Edition (DSM-5), encopresis involves repeated defecation in inappropriate places, occurring at least once per month for a minimum duration of three months, in children at least four years of age [1]. The global prevalence is estimated to range from 0.8% to 7.8%, varying depending on the diagnostic criteria and the population studied [1]. While episodes typically occur during the day, the presence of isolated nocturnal encopresis should prompt consideration of underlying organic causes [1]. This disorder is often associated with a considerable psychological burden, not only for the affected child but also for the family unit [2]. In the United States, functional encopresis affects approximately 4.1% of children aged five to six years, with the prevalence decreasing to 1.6% among those aged 11 to 12 years [3]. Most medical consultations occur around the age of seven to eight years, indicating that families often seek help when the symptoms persist and begin to interfere with the child's social functioning and emotional well-being [4]. Although less frequent, cases of encopresis have been reported in early adulthood [5]. 

## Materials and methods

This retrospective, descriptive study was conducted between January 2021 and December 2024 in the pediatric outpatient gastroenterology clinic of Mohammed VI University Hospital in Oujda, Morocco. Data were collected through the HOSIX hospital software system (SIVSA, Spain). Inclusion criteria were children aged four years or older, with prior achievement of toilet training, diagnosed with encopresis regardless of etiology, and followed during outpatient consultations within the study period. Patients with incomplete medical records, known psychiatric disorders unrelated to encopresis, or younger than four years at diagnosis were excluded.

Collected data included age at symptom onset, type of encopresis (with or without constipation), associated symptoms, duration before treatment initiation, urban or rural origin, presence of underlying neurological or congenital conditions, and therapeutic management details. Variables were extracted directly from medical records without standardized scales or assessment tools due to the retrospective design.

Data quality was maintained through a systematic review of medical files within the hospital software. Missing data were handled by excluding incomplete records from the analysis. Descriptive statistical analysis was performed using Microsoft Excel (Microsoft Corp., Redmond, United States), presenting frequencies and percentages for categorical variables, and means or medians for continuous variables. Ethical approval was obtained, and patient confidentiality was maintained via anonymization in accordance with the ethical guidelines.

## Results

The study population presented with an average age of five years at the onset of encopresis symptoms, contrasting with an average age of four years for the achievement of initial bowel control. A subset of the cases, representing 20% (N = 4) of the total, exhibited symptom onset between the ages of four and five years. The majority of cases, comprising 80% (N = 16) of the study population, experienced a later onset of symptoms, beginning after five years of age. The study did not identify any instances of primary encopresis within the analyzed cohort.

**Figure 1:**
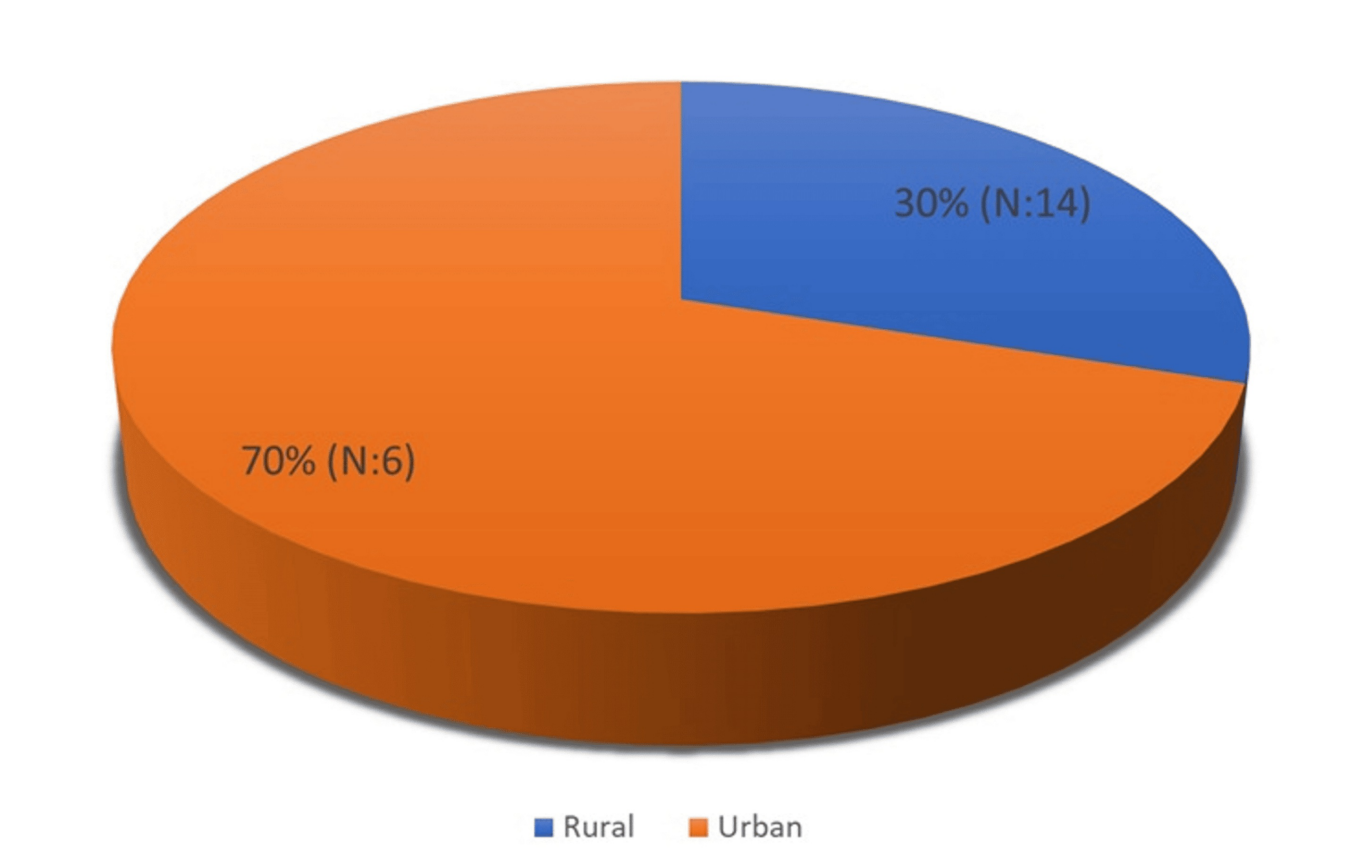
Distribution of patients by residence

The demographic distribution of the study participants revealed that 70% (N = 14) resided in urban environments, while 30% (N = 6) were from rural areas (Figure 1).

The primary reason for the consultation was chronic constipation, which was reported in 85% (N = 17) of the cases. Anal incontinence, without concurrent constipation, was reported as the sole symptom in 10% (N = 2) of the cases. A small proportion of the study population, specifically 5% (N = 1) of the children, presented with a combination of anal and urinary incontinence.

In the majority of cases, specifically 85% (N = 17), encopresis was determined to be a direct consequence of chronic constipation, resulting in prolonged fecal impaction. A single case (N = 1) of congenital megacolon was identified as an underlying etiology. Neurological involvement was observed in 10% (N = 2) of the cases. These cases included one instance of centromedullary syringomyelia, diagnosed via magnetic resonance imaging (MRI) of the spinal cord, and one case of acute myelitis identified within this subset (Table 1).

**Table 1:** Therapeutic care of patients PEG: Polyethylene glycol

Treatment	Number (n)	Percentage (%)
PEG-based laxatives	20	100%
Additional osmotic laxatives	6	30%
Hygiene and dietary modifications	20	100%
Education and toilet training support	20	100%
Perineal rehabilitation	8	40%

The therapeutic approach implemented involved a combined strategy. This included the use of polyethylene glycol (PEG)-based laxatives, occasionally supplemented with other osmotic laxatives. The treatment regimen also incorporated hygiene and dietary modifications, emphasizing a fiber-rich diet, adequate hydration, and the establishment of regular toilet routines. Additionally, patient and caregiver education, as well as perineal rehabilitation, were integral components of the therapeutic intervention (Table 2).

**Table 2:** The etiologies of encopresis found in patients

Etiology	Number (n)	Percentage (%)
Functional encopresis due to chronic constipation	17	85%
Congenital megacolon	1	5%
Neurological causes (total)	2	10%
– Centromedullary syringomyelia	1	5%
– Acute myelitis	1	5%

## Discussion

Our study provides significant insights into the epidemiological, clinical, and therapeutic characteristics of encopresis, while highlighting certain aspects that both align with and diverge from existing literature. Firstly, the average age of symptom onset in our cohort was five years, which is consistent with most published data, where encopresis is typically diagnosed after the age of four [6].

This agreement reinforces the robustness of this age range as a critical period for the emergence of symptoms. However, a notable divergence arises from the high prevalence of 80% (N = 16) of cases in our study that began after the age of five years. This finding contrasts with other studies reporting a more uniform distribution of ages at onset [4,7]. Several hypotheses can be proposed to explain this discrepancy. Cultural or familial differences in expectations and practices surrounding toilet training may delay both the recognition of symptoms and medical consultation. Additionally, in certain sociocultural contexts, parents may tolerate or underreport fecal incontinence for extended periods, particularly in the absence of associated behavioral issues. These factors could contribute to delayed diagnoses, explaining the predominance of late-onset cases in our sample.

In line with the literature, which describes an average age of onset around five years in clinical populations [7], our results reinforce this temporal reference. However, our study revealed an absence of primary encopresis cases, which is a significant observation. While some studies report primary encopresis in up to 10% (N = 2) of cases, our cohort exclusively consisted of secondary encopresis [5]. This may reflect successful early toilet training within the studied population or result from sampling bias, perhaps because primary cases are less likely to seek care or are misclassified by caregivers or primary care providers. Alternatively, this could suggest that children with primary encopresis are managed differently or are underrepresented in hospital settings.

Another interesting epidemiological aspect is the predominance of urban origin among patients, with 70% (N = 14) residing in urban areas. This finding aligns with previous studies [4] and may reflect greater awareness, earlier screening, or increased healthcare utilization among urban families. Urban environments may also expose children to greater psychological or social pressures regarding continence and dietary habits that predispose them to constipation. In contrast, the 30% (N = 6) of cases from rural areas may underestimate the actual prevalence due to diagnostic delays, limited access to specialized care, or cultural differences in interpreting bowel habits.

From an etiological perspective, chronic constipation emerged as the primary cause of encopresis in our sample, accounting for 85% (N = 17) of cases. This finding closely aligns with previous studies attributing 80% to 95% of encopresis cases to functional constipation and fecal retention [8]. These figures underscore the central role of constipation in the pathophysiology of encopresis and justify prioritizing laxative-based therapeutic protocols. Notably, the average delay of 12 months between symptom onset and treatment initiation reflects a common challenge also noted in the literature [7]. This prolonged delay may stem from parental misconceptions about the condition, stigma, or underestimation of its severity, highlighting the need for early education and intervention strategies. 

In contrast to other studies reporting a higher proportion of organic causes, our study identified only one case of congenital megacolon. This lower prevalence may be attributed to exclusion criteria or referral patterns. Interestingly, neurological causes were identified in 10% (N = 2) of cases, a significant proportion that included rare conditions such as centromedullary syringomyelia and acute myelitis [9]. This finding emphasizes the importance of thorough clinical evaluation, especially in cases that do not respond to conventional treatment. 

Therapeutically, our approach, which combines PEG-based laxatives, dietary and hygienic measures, and family education, aligns with current recommendations and guidelines [8]. The generally favorable outcomes observed reinforce the effectiveness of this standardized strategy. However, we note that more advanced behavioral techniques, such as biofeedback therapy, although not utilized in our study, have shown promise in other research. Integrating such interventions into future therapeutic protocols could potentially improve outcomes, particularly for refractory cases [10].

Our results largely align with those in the literature, particularly regarding age of onset, urban predominance, and the predominance of constipation as an etiological factor. However, certain divergences, such as late presentation, absence of primary encopresis, and notable neurological involvement, provide valuable insights that may inform local diagnostic and therapeutic strategies. These findings confirm the importance of a multifactorial and individualized approach to managing encopresis in clinical practice.

Nevertheless, our study has several limitations that must be acknowledged. The relatively small sample size restricts the generalizability of our findings and limits the statistical power to detect less common associations. As a retrospective study relying on clinical records and parental reports, there is a potential for recall bias and incomplete data capture, especially regarding behavioral symptoms and comorbidities. The absence of standardized psychiatric or neurodevelopmental assessments, such as for anxiety, attention deficit hyperactivity disorder (ADHD), or autism spectrum disorders, may have led to underrecognition of important comorbid factors influencing encopresis. Moreover, data extraction and quality control relied on the hospital software system (HOSIX), which contains detailed, longitudinal patient data accessible only to authorized pediatric clinicians, supporting data accuracy.

Future prospective studies with larger cohorts, standardized diagnostic tools, and longer follow-up are needed to better understand the multifactorial etiology and optimize management strategies for encopresis across diverse populations.

## Conclusions

Encopresis represents a significant challenge for affected children and their families. While diagnosis is typically achieved through clinical assessment, effective management requires a comprehensive approach. First-line treatment emphasizes collaborative behavioral interventions involving parents and caregivers, coupled with the judicious use of oral osmotic laxatives, often at higher doses and for extended durations. When these initial measures prove insufficient, further interventions may be considered, although their efficacy remains less definitively established. Despite the complexities of treatment, the long-term prognosis for encopresis is generally favorable. However, a substantial proportion of children may require ongoing laxative support. Recognizing the importance of early intervention, prompt diagnosis and treatment significantly improve the likelihood of successful and lasting recovery, minimizing the potential for long-term physical and emotional sequelae.
